# Epigenetic Bridge Between Oxidative Balance of Koreans and TCGA Pan-Cancer Risk: Sex-Specific DNA Methylation Signatures

**DOI:** 10.3390/antiox15030386

**Published:** 2026-03-19

**Authors:** Sun-Young Kang, Jeong-Soo Gim, Hyunbin Jo, Jeong-An Gim

**Affiliations:** 1 Institute for Molecular Metabolism Innovation, Soonchunhyang University, Asan 31538, Republic of Korea; 2 Department of Pet Health Care, Busan Health University, Busan 49318, Republic of Korea; 3 Department of Companion Animal Health, Tongmyong University, Busan 48520, Republic of Korea; 4 Department of Medical Science, Soonchunhyang University, Asan 31538, Republic of Korea

**Keywords:** oxidative balance score, epigenetics, DNA methylation, pan-cancer, KoGES, TCGA

## Abstract

Oxidative stress is a hallmark of carcinogenesis, yet the epigenetic mechanisms linking the lifestyle-based Oxidative Balance Score (OBS) to cancer risk remain poorly understood. This study investigated the epigenetic bridge between OBS and pan-cancer susceptibility using a multi-cohort approach integrating population-based and cancer genomic data. We calculated OBS based on 16 dietary and lifestyle factors (including dietary fiber, vitamins, minerals, physical activity, smoking, alcohol, and BMI) for 2749 participants from the Korean Genome and Epidemiology Study (KoGES) and identified OBS-associated CpG sites via epigenome-wide association analysis. These markers were validated against The Cancer Genome Atlas (TCGA) pan-cancer dataset using a novel Hybrid Pi-score (HyPi) to quantify the directional consistency between OBS-driven methylation in healthy individuals and cancer-specific epigenetic alterations across three clinical comparisons: normal vs. tumor, survival outcomes, and tumor stage. We observed profound sex-specific epigenetic signatures, with zero overlap in the top 200 OBS-associated CpG sites between males and females, underscoring fundamental sexual dimorphism in oxidative stress-epigenome interactions. Notably, the top 20 OBS-associated CpGs demonstrated strong directional consistency with multiple cancer types in TCGA, particularly in kidney renal clear cell carcinoma and lung adenocarcinoma, exhibiting methylation patterns inversely correlated with tumorigenesis. Mechanistically, these findings support the role of one-carbon metabolism and vitamin C-dependent DNA demethylation pathways in mediating OBS effects. Our study provides the first evidence of an epigenetic link between lifestyle-based oxidative balance and pan-cancer risk, highlighting the utility of the HyPi score as a novel sex-specific predictive biomarker for cancer prevention. These results suggest that optimizing oxidative balance through precision nutrition may epigenetically modulate cancer susceptibility, opening new avenues for personalized prevention strategies.

## 1. Introduction

Oxidative stress, defined as an imbalance between reactive oxygen species (ROS) production and antioxidant defenses [1], plays a pivotal role in the initiation and progression of cancer [2,3,4]. ROS function as pleiotropic physiological signaling agents but can cause cellular damage when dysregulated [1].

While individual antioxidants have been extensively studied [5,6], the Oxidative Balance Score (OBS) offers a comprehensive measure of an individual’s oxidative status by integrating multiple dietary and lifestyle factors [7,8,9,10,11]. Age-related changes in body composition and metabolic function [7] may influence oxidative balance, making OBS particularly relevant for assessing cancer risk across the lifespan. Notably, oxidative balance and antioxidant capacity exhibit pronounced sex-specific differences driven by hormonal status, body composition, and lifestyle patterns, suggesting that the epigenetic impact of OBS on cancer risk may differ between males and females.

This composite approach has demonstrated associations with colorectal cancer risk and other chronic diseases [8,9], as well as metabolic conditions such as kidney stone formation [12]. However, the molecular mechanisms—specifically epigenetic alterations—through which OBS influences cancer risk in a healthy population remain elusive. In particular, it remains unknown whether OBS-associated DNA methylation patterns in the general population are directionally concordant with those observed in high-risk or malignant tissues.

DNA methylation is a fundamental epigenetic mechanism that regulates gene expression [13] and is frequently dysregulated in cancer development [14]. Aberrant DNA methylation patterns, including global hypomethylation and gene-specific hypermethylation, are hallmarks of malignant transformation [14,15]. Importantly, DNA methylation can be influenced by environmental and dietary factors [16,17], providing a mechanistic link between lifestyle exposures and cancer risk.

This study utilizes a multi-cohort approach, combining population-based DNA methylation data from KoGES with pan-cancer epigenomic profiles from TCGA, to determine whether systemic oxidative balance leaves epigenetic imprints that mirror cancer-associated methylation patterns.

## 2. Materials and Methods

### 2.1. Study Population

We utilized human DNA methylation data from the Korean Genome and Epidemiology Study (KoGES), specifically the Ansan-Ansung (ASAS) and City (CITY) cohorts. The KoGES consortium is a large-scale prospective cohort study designed to investigate genetic and environmental determinants of chronic diseases in the Korean population [18]. The dataset comprised 2749 individuals (ASAS: 2001 baseline and 2009 follow-up; CITY: 2004 baseline). This study was conducted with bioresources from National Biobank of Korea, the Korea Disease Control and Prevention Agency, Cheongju, Republic of Korea (KBN-2024-045). This study was conducted in accordance with the Declaration of Helsinki with the approval of Soonchunhyang University Institutional Review Board (2024-05-049). DNA methylation was profiled using the Illumina Infinium HumanMethylation450 BeadChip (San Diego, CA, USA). Beta-values were normalized and filtered for quality control.

### 2.2. Calculation of Oxidative Balance Score (OBS)

To comprehensively assess the oxidative stress status of the study population, we calculated the Oxidative Balance Score (OBS) by integrating 16 dietary and lifestyle factors. These components were classified into antioxidants (11 factors) and pro-oxidants (5 factors) based on their established biological roles in oxidative stress modulation. Two documents were primarily referenced for this OBS calculation. First, the paper that first introduced OBS calculations and calculated the antioxidant and pro-oxidant components for each variable was referenced [8]. Similarly, the most recent two papers on OBS calculations were referenced [10,11], and calculations were performed according to the KoGES variables, as described below.

#### 2.2.1. Assignment of Scores

We employed a sex-specific tertile-based scoring system. For each component, participants were stratified into three groups (Tertile 1 [Low; bottom 33%], Tertile 2 [Intermediate 33%], Tertile 3 [High; top 33%]) based on the distribution of data within their respective sex.

#### 2.2.2. Antioxidant Components (Positive Scoring)

This category included 10 dietary nutrients (Dietary Fiber, Carotene, Riboflavin, Niacin, Vitamin B6, Total Folate, Vitamin C, Vitamin E [α-tocopherol equivalents], Calcium, Zinc) and 1 lifestyle factor (Physical Activity). Higher intake or activity levels were assigned higher scores to reflect their protective effects against oxidative stress.

Score Assignment:

- Tertile 1 (Lowest intake/activity): 0 points.
- Tertile 2 (Intermediate): 1 point.
- Tertile 3 (Highest intake/activity): 2 points.

#### 2.2.3. Pro-Oxidant Components (Reverse Scoring)

This category included 2 dietary nutrients (Total Fat, Iron) and 3 lifestyle factors (Smoking Status, Body Mass Index [BMI], Alcohol Consumption). Higher levels of these factors were considered detrimental to oxidative balance and were assigned lower scores.

Standard Reverse Scoring (Fat, Iron, BMI):

- Tertile 1 (Lowest intake/BMI): 2 points.
- Tertile 2 (Intermediate): 1 point.
- Tertile 3 (Highest intake/BMI): 0 points.

Categorical Scoring (Smoking):

- Never Smoker: 2 points.
- Former Smoker: 1 point.
- Current Smoker: 0 points.

Special Scoring for Alcohol Consumption: Given the nonlinear distribution of alcohol consumption (excessive zero-inflation), we applied a modified scoring algorithm to distinctively weigh non-drinkers and drinkers.

- Non-drinkers (0 g/day): 2 points.
- Light/Moderate Drinkers (Lower 50% of non-zero values): 1 point.
- Heavy Drinkers (Upper 50% of non-zero values): 0 points.

#### 2.2.4. Final Score Summation

The final OBS was calculated by summing the scores of all 16 components. The theoretical range of the OBS was 0 to 31 points, with higher scores indicating a predominance of antioxidant exposure (favorable oxidative balance).

### 2.3. TCGA Pan-Cancer Methylation Analysis and Pi-Score Calculation

#### 2.3.1. DMR Selection by Three Comparisons

To investigate the epigenetic relevance of OBS in cancer, we utilized DNA methylation data from The Cancer Genome Atlas (TCGA) Pan-Cancer Atlas. The TCGA represents the most comprehensive multi-platform analysis of cancer genomics, encompassing 33 tumor types with integrated molecular profiling [19,20]. Cell-of-origin patterns have been shown to dominate molecular classification across these diverse cancer types [19], with specific insights available for renal cell carcinoma [21] and lung adenocarcinoma [22]. We focused on three key clinical comparisons to identify Differentially Methylated Regions (DMRs):

Normal vs. Tumor (NT): Comparing adjacent normal tissue with primary tumor tissue.

Survival Analysis (Survival): Comparing patients who succumbed to the disease (Dead) vs. those who survived (Alive).

Tumor Stage (Stage): Comparing early-stage (Stage I/II) vs. late-stage (Stage III/IV) tumors.

#### 2.3.2. Calculation of the Pi-Score (π)

To simultaneously capture both the statistical significance and the magnitude of methylation changes in cancer, we calculated the Pi-score (π) for each CpG site in the TCGA dataset. The π-score is defined as the product of the negative logarithm of the *p*-value and the fold change (mean difference), derived from differential methylation analysis.

π = −log10(*p*-value) × (Fold change)

### 2.4. Epigenome-Wide Association and Hybrid Pi-Score Analysis

We performed an Epigenome-Wide Association Study (EWAS) within the KoGES cohort to identify CpG sites associated with OBS and subsequently evaluated their directional consistency with TCGA cancer signatures using a Hybrid Pi-score approach. The analytical framework incorporated principles of differential variability analysis, which has been demonstrated to improve identification of cancer risk markers in DNA methylation studies [23,24].

All statistical analyses and visualizations were performed using R statistical software (version 4.5.1) [25], utilizing packages including dplyr, ggplot2, and custom functions for score generation.

#### 2.4.1. OBS-Methylation Association (KoGES)

Linear regression models were fitted for each CpG site to estimate the association between OBS and methylation levels (β-values), adjusting for potential confounders such as age, sex, and white blood cell composition.

#### 2.4.2. Correlation Analysis and Calculation of the Hybrid Pi-Score (HyPi)

We calculated Pearson (r) and Spearman (ρ) correlation coefficients between the KoGES OBS and value. Then, to quantify the “epigenetic impact potential” of OBS in the general population comparable to the cancer-derived *π*-score, we derived the Hybrid Pi-score (HyPi) from the KoGES EWAS results [23]. The HyPi integrates the statistical significance of the OBS association with the direction of the effect:

HyPi=(CRpearson×−log10PVpearson)+(CRspearman×−log10PVspearman)

Data manipulation and visualization were conducted using tidyverse packages [26]. DNA methylation preprocessing utilized minfi and related Bioconductor packages [27].

#### 2.4.3. Quad-Plot Analysis

We visualized the relationship using scatter plots divided into four quadrants.

- Consistent (+/+): HyPi > 0 and π > 0 (OBS increases methylation, cancer increases methylation).
- Consistent (−/−): HyPi < 0 and π < 0 (OBS decreases methylation, cancer decreases methylation).
- Inconsistent (+/− or −/+): Direction of effect differs between OBS and cancer.

## 3. Results

### 3.1. Distinct Sexual Dimorphism in OBS-Associated Epigenetic Signatures

To determine whether oxidative balance influences the epigenome differently in males and females, we first identified the top 200 CpG sites most strongly associated with OBS in the KoGES cohort (HyPi score). A Venn diagram analysis revealed a striking lack of overlap between the sexes, with zero shared CpG sites among the top 200 markers (Supplementary Materials, Figure S1). This suggests that the epigenetic mechanisms responding to oxidative balance are inherently sex-specific.

**Figure 1:**
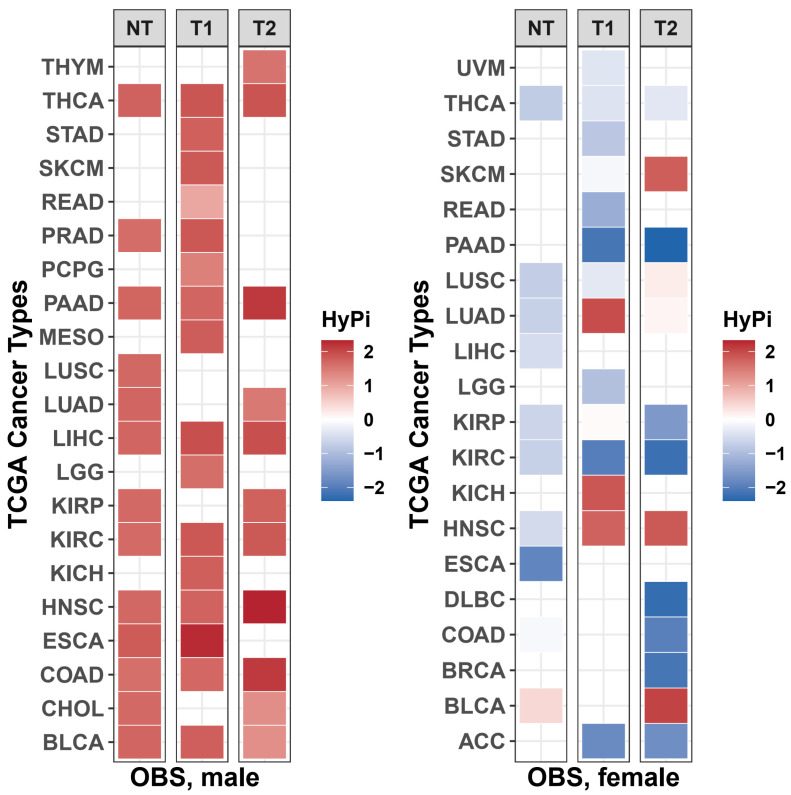
Global heatmap of Oxidative Balance Score (OBS) effects across TCGA pan-cancer types. Heatmaps representing the Hybrid Pi-score (HyPi) landscape for males (left panel) and females (right panel). The *y*-axis lists the TCGA cancer type abbreviations (e.g., THYM, THCA, STAD), and the *x*-axis represents the three clinical comparison categories: Normal vs. Tumor (NT), Survival/Dead vs. Alive (T1), and Tumor Stage I/II vs. III/IV (T2). The color gradient ranges from blue (negative association) to red (positive association), reflecting the direction and magnitude of the epigenetic link between OBS and cancer features.

This sexual dimorphism was further corroborated by comparing the HyPi scores across the TCGA pan-cancer datasets. When the cumulative effect of OBS (Total HyPi × Pi Score) was plotted for males versus females, the data points significantly deviated from the diagonal line (y = x), which represents shared mechanisms (Supplementary Materials, Figure S2). The scatter mainly occupied the off-diagonal spaces, indicating that specific cancer types (e.g., KIRC, LUAD) exhibit differential epigenetic sensitivity to oxidative balance depending on biological sex. Then, global heatmap of OBS effects across TCGA pan-cancer types (Figure 1).

### 3.2. Identification of OBS-Driven ‘Master’ CpG Sites

We identified the top 20 “Master” CpG sites that exhibited the highest Hybrid Pi-scores (HyPi), representing markers with both high statistical significance and large effect sizes in response to OBS. The methylation levels of all 20 CpG sites were visualized as heatmap (Supplementary Materials, Figure S3).

**Figure 2:**
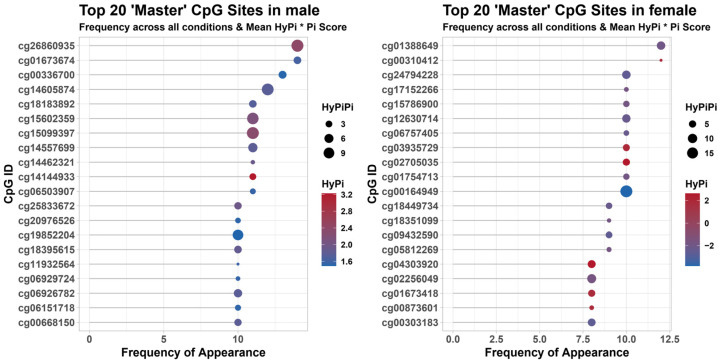
Identification of Top 20 ‘Master’ CpG sites associated with OBS. Bubble plots ranking the top 20 CpG sites based on their association with OBS in males (**left**) and females (**right**). The *y*-axis displays the specific CpG identifiers (cg#), and the *x*-axis represents the frequency of their appearance across bootstrap or model iterations. The size of each bubble corresponds to the magnitude of the HyPi score, and the color gradient (blue to red) indicates the specific HyPi value. These markers represent the most robust epigenetic targets modulated by oxidative balance.

In males (Figure 2, Left), the identified CpG sites showed a high frequency of appearance across multiple analytical models, with HyPi values indicating robust hypermethylation or hypomethylation tendencies associated with higher OBS.

In females (Figure 2, Right), a completely distinct set of CpG sites was identified. The bubble plots illustrate that while some CpGs appear frequently, their effect sizes (indicated by color intensity) vary, highlighting the complexity of the female epigenetic response to oxidative stress modulators. These ‘Master’ CpGs serve as potential sex-specific biomarkers for monitoring oxidative balance.

### 3.3. Directional Consistency Between OBS and Pan-Cancer Risk

A critical objective of this study was to validate whether OBS-induced methylation changes in a healthy population (KoGES) predict cancer-associated methylation patterns (TCGA). We utilized Quad-plots to visualize the correlation between the KoGES HyPi score (*x*-axis) and the TCGA Pi-score (*y*-axis) across three clinical comparisons: Normal vs. Tumor (NT), Survival (T1), and Stage (T2).

**Figure 3:**
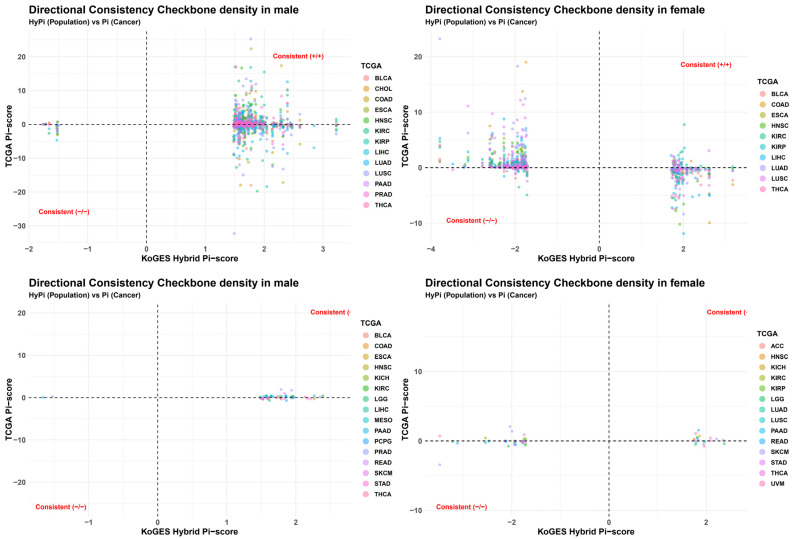

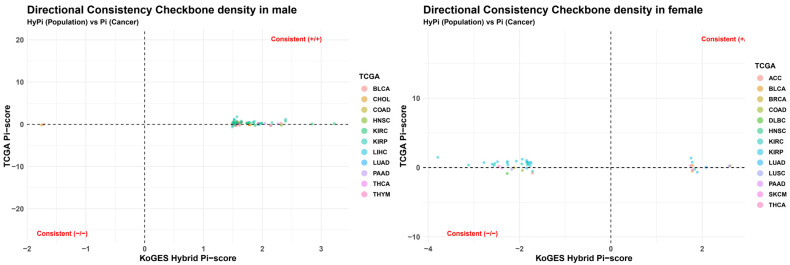
Directional consistency check between KoGES population data and TCGA cancer data. Quad-plots evaluating the directional alignment of methylation changes. The *x*-axis represents the KoGES Hybrid Pi-score (effect of OBS in the healthy population), and the *y*-axis represents the TCGA Pi-score (methylation difference in cancer). (**Left**) Normal vs. Tumor (NT) comparison. (**Right**) Survival (T1) comparison. Tumor Stage (T2) comparison. Quadrants labeled “Consistent (+/+)” (top-right) and “Consistent (−/−)” (bottom-left) indicate CpG sites where OBS-driven methylation changes mimic the patterns observed in cancer, suggesting a potential predictive value. The colored points represent different cancer types.

As shown in Figure 3, a significant proportion of CpGs clustered in the “Consistent (−/−)” and “Consistent (+/+)” quadrants.

Consistent (−/−): A negative HyPi indicates that higher OBS reduces methylation, and a negative TCGA Pi indicates that cancer reduces methylation. This alignment suggests that OBS may influence genes that are typically dysregulated in cancer.

Consistent (+/+): Conversely, positive consistency implies that high OBS increases methylation in regions that are also hypermethylated in cancer.

The density of points in the consistent quadrants was particularly pronounced in the Survival (T1) and Normal vs. Tumor (NT) comparisons, suggesting that the epigenetic footprint of oxidative balance is relevant to both carcinogenesis and prognosis.

### 3.4. Pan-Cancer Epigenetic Landscape and Clinical Linkages

We further explored how these OBS-associated signatures map across different cancer types.

Global Contribution: The variable contribution analysis (Supplementary Materials, Figure S4) revealed that certain cancer types, such as Kidney Renal Clear Cell Carcinoma (KIRC) and Lung Adenocarcinoma (LUAD), contributed disproportionately to the total epigenetic signal derived from OBS markers. This trend was consistent across NT, T1, and T2 comparisons but varied in magnitude between sexes.

**Figure 4:**
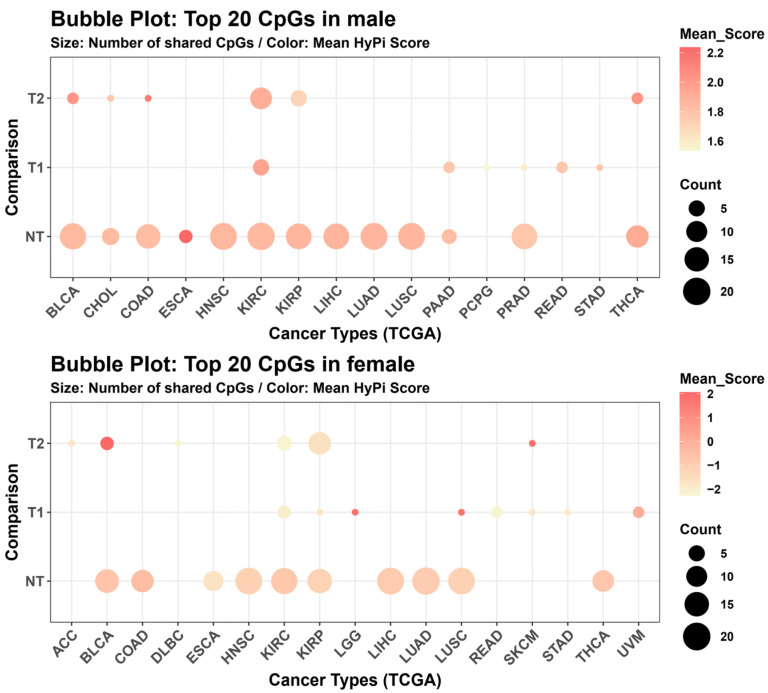
Bubble plot distribution of Top 20 CpGs across cancer types. Bubble plots mapping the distribution of the top 20 CpGs across TCGA cancer types (*x*-axis) and comparison categories (*y*-axis). (**Top**) Male cohort. (**Bottom**) Female cohort. The size of the bubbles represents the count of significant CpGs found in that specific cancer/comparison intersection, while the color indicates the mean HyPi score. This visualizes the “hotspots” of OBS-related epigenetic activity across the pan-cancer landscape.

We further mapped the global distribution of these OBS-associated epigenetic signatures across the TCGA pan-cancer landscape using a secondary bubble plot analysis (Figure 4). Here, the *x*-axis represents the diverse cancer types (e.g., KIRC, LUAD, BRCA), and the *y*-axis represents the three clinical comparison categories: Normal vs. Tumor (NT), Survival (T1), and Tumor Stage (T2). The bubble size indicates the count of significant OBS-associated CpGs found within that specific intersection, serving as a proxy for “epigenetic density.” The color intensity represents the mean HyPi score of those CpGs. The analysis highlighted specific “epigenetic hotspots.” For instance, large, intensely colored bubbles were observed in metabolic cancers such as Kidney Renal Clear Cell Carcinoma (KIRC) and respiratory cancers like Lung Adenocarcinoma (LUAD), particularly in the NT comparison. This suggests that the epigenetic impact of oxidative balance is not uniform but is disproportionately concentrated in specific cancer types and is most influential in distinguishing normal tissue from tumor tissue.

**Figure 5:**
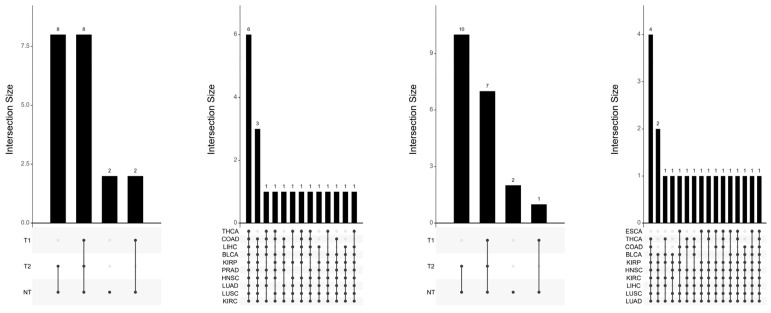
Upset plot and intersection analysis of OBS markers. A visualization of the intersections between sets of significant CpG sites across different clinical comparisons (NT, T1, T2). The vertical bars represent the size of the intersection (number of shared CpGs), while the matrix dots below indicate the specific combination of sets being compared. This figure highlights whether OBS-associated markers are unique to specific clinical outcomes (e.g., survival only) or shared across multiple cancer features.

To dissect the clinical utility and functional overlap of the identified epigenetic markers, we employed an UpSet plot analysis (Figure 5). Unlike traditional Venn diagrams, which can be cluttered with multiple sets, the UpSet plot provides a clear matrix-based visualization of the intersections between significant CpG sets across the three clinical dimensions: Normal vs. Tumor (NT), Survival (T1), and Tumor Stage (T2). Interpretation of Intersections: The vertical bars represent the size of the intersection (i.e., the number of shared CpGs), while the connected dots in the matrix below indicate which specific sets are being compared. The analysis revealed that the majority of OBS-associated CpGs were unique to the Normal vs. Tumor (NT) comparison, indicated by the tallest isolated bar. However, a significant subset of markers formed an intersection between NT and Survival (T1) comparisons. This specific overlap suggests that oxidative balance modulates a core set of genes that are not only involved in the initial neoplastic transformation (diagnosis) but also play a persistent role in determining patient prognosis (survival). The minimal overlap with Stage (T2) suggests that OBS-driven epigenetics may be an early event in carcinogenesis rather than a driver of late-stage progression.

**Figure 6:**
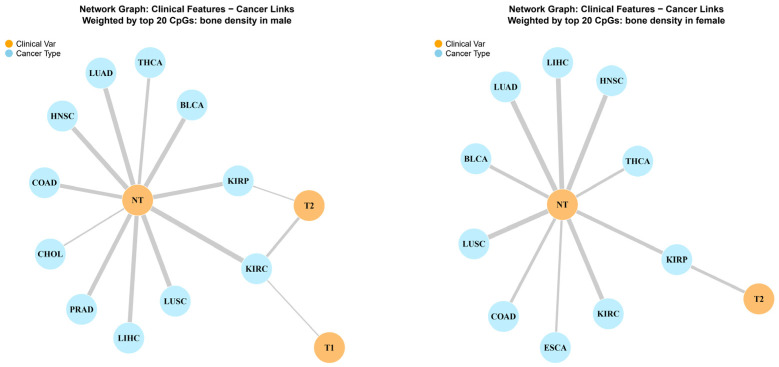
Network graph of clinical features and cancer links weighted by Top 20 CpGs. Network diagrams illustrating the connectivity between clinical variables (orange nodes: NT, T1, T2) and cancer types (blue nodes), weighted by the strength of the top 20 OBS-associated CpGs. (**Left**) Male network: Shows strong centralization around the NT node, indicating OBS markers primarily distinguish tumor from normal tissue. (**Right**) Female network: Displays a more distributed connectivity, suggesting a broader range of clinical implications.

Network analysis visualized the links between clinical features (Comparison types) and specific cancer types, weighted by the top 20 CpGs (Figure 6). In males, the network showed a dense clustering of links around the ‘NT’ node, suggesting that OBS markers are most effective at distinguishing normal from tumor tissues. In females, the network displayed a broader distribution, implicating a wider range of cancer types.

**Figure 7:**
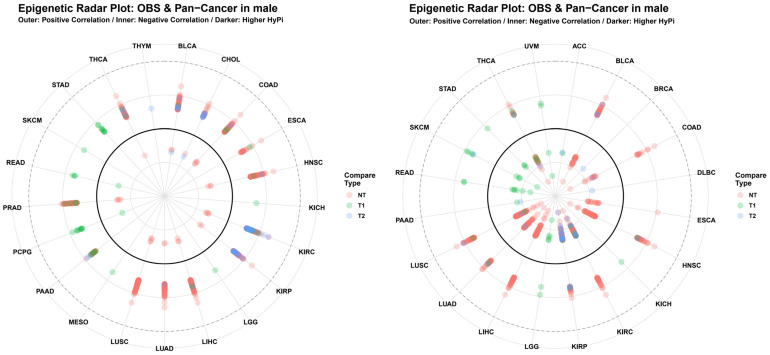
Epigenetic Radar Plot of OBS versus Pan-Cancer. Circular radar plots visualizing the magnitude of the Hybrid Pi-score across the pan-cancer spectrum for males. The outer labels represent the different TCGA cancer types. The radial distance from the center represents the HyPi score, with separate lines/colors for NT, T1, and T2 comparisons. This multidimensional view allows for the rapid identification of cancer types that exhibit extreme epigenetic sensitivity to oxidative balance (outliers on the radar).

Multidimensional Profiling: The Genetic Radar Plots (Figure 7) provided a holistic view of the HyPi scores across all analyzed cancer types. The distortion of the radar rings highlights that OBS does not affect all cancers equally; rather, it exhibits specific “epigenetic tropism” toward metabolic and respiratory cancers.

## 4. Discussion

### 4.1. The Epigenetic Bridge: Linking Oxidative Balance to Pan-Cancer Risk

In this study, we successfully established an epigenetic bridge connecting the lifestyle-based Oxidative Balance Score (OBS) to pan-cancer susceptibility. By integrating large-scale population data (KoGES) with comprehensive cancer genomic profiles (TCGA), we demonstrated that oxidative balance is not merely a transient metabolic state but leaves a lasting “epigenetic footprint” on the genome. Our novel Hybrid Pi-score (HyPi) approach allowed us to quantify this relationship, revealing that the cumulative epigenetic impact of antioxidant-rich behaviors can modulate specific genomic loci frequently dysregulated in carcinogenesis. To our knowledge, this is the first study to utilize a hybrid scoring system to validate the directional consistency of OBS-associated methylation patterns against a pan-cancer atlas. Importantly, this epigenetic bridge does not imply a direct causal pathway but rather reflects a molecular concordance between lifestyle-associated epigenetic variation in healthy individuals and cancer-associated epigenetic dysregulation.

### 4.2. Sexual Dimorphism in Epigenetic Regulation

One of the most striking findings of our study is the complete absence of overlap in the top 20 OBS-associated CpG sites between males and females (Supplementary Materials, Figure S1). This points to a profound sexual dimorphism in how oxidative stress influences the epigenome, consistent with well-documented sex differences in cancer incidence, outcomes, and therapeutic responses [28,29]. Previous research has characterized substantial sex-specific differences in DNA methylation patterns across the autosomal genome [30], suggesting that biological sex fundamentally shapes epigenetic architecture. Although the identified CpG sites are located on autosomal chromosomes, accumulating evidence suggests that sex hormones and sex-specific transcriptional programs can indirectly shape autosomal methylation patterns through differential regulation of epigenetic enzymes and chromatin accessibility.

Biologically, this dimorphism can be attributed to the complex interplay between sex hormones and oxidative stress defense mechanisms [31]. Estrogens are known to possess antioxidant properties and can regulate the expression of antioxidant enzymes such as superoxide dismutase (SOD) and glutathione peroxidase (GPx) [31]. Furthermore, sex hormones can directly influence the activity of DNA methyltransferases (DNMTs), creating distinct epigenetic landscapes in males and females [30]. Our results suggest that the “epigenetic machinery” responding to dietary and lifestyle oxidative factors operates through fundamentally distinct pathways in men and women. Consequently, precision nutrition and cancer prevention strategies targeting oxidative balance must be fundamentally sex-specific.

The divergence observed in the global scatter plots (Supplementary Materials, Figure S2) further reinforces that the epigenetic susceptibility to cancer under varying oxidative conditions differs markedly by sex, with implications for personalized medicine approaches [28,29].

### 4.3. Directional Consistency: Vulnerability vs. Protection

The Quad-plot analysis (Figure 6) provided critical insights into the nature of the OBS-cancer relationship. We observed a significant clustering of CpG sites in the “Consistent” quadrants (both +/+ and −/−).

Interpretation of Consistency: The fact that OBS-driven methylation in healthy individuals (KoGES) aligns with methylation patterns in cancer (TCGA) suggests that these specific loci are “oxidative stress-sensitive hotspots.” These regions are highly plastic and responsive to environmental redox states. Importantly, directional consistency should not be interpreted as uniformly protective or deleterious. Rather, it reflects shared epigenetic sensitivity to oxidative balance, where the biological consequence likely depends on genomic context, cell type, and disease stage.

Mechanism of Action: For example, in the “Consistent (−/−)” quadrant, high OBS (favorable antioxidant status) was associated with hypomethylation, which mirrored the hypomethylation observed in specific tumor contexts. While global hypomethylation is a hallmark of cancer (often leading to genomic instability), gene-specific hypomethylation can activate tumor suppressors. Conversely, if high OBS induces hypermethylation in loci that are also hypermethylated in cancer (Consistent +/+), it raises complex questions about whether OBS is capturing a compensatory mechanism or if certain “pro-oxidant” signatures are paradoxically retained.

The strong connectivity in the Normal vs. Tumor (NT) network (Figure 6) for males specifically suggests that OBS-related markers are potent classifiers for distinguishing healthy tissue from neoplastic transformation, validating the utility of HyPi as a predictive biomarker. These findings align with principles of differential variability analysis [23], where epigenetic markers with high discriminatory power can identify precursor lesions and early cancer development.

### 4.4. Pan-Cancer Associations: Metabolic and Respiratory Links

Our pan-cancer analysis highlighted that OBS-associated epigenetic markers were disproportionately linked to specific cancer types, most notably Kidney Renal Clear Cell Carcinoma (KIRC) and Lung Adenocarcinoma (LUAD).

Kidney Cancer (KIRC): The kidney is a metabolically active organ highly susceptible to oxidative damage due to its filtration function. The strong association suggests that dietary antioxidants may play a crucial role in preserving the epigenetic integrity of renal tissues.

Lung Cancer (LUAD): Given that smoking is a major component of the pro-oxidant score in our OBS calculation, the strong link to lung cancer is biologically plausible. However, the epigenetic connection persists even when considering the composite score, indicating that the balance of antioxidants can mitigate some smoking-induced epigenetic alterations.

The Genetic Radar Plots (Figure 7) clearly visualized this “epigenetic tropism,” showing that the impact of OBS is not uniform across all cancers but is concentrated in organs with high oxygen consumption and metabolic turnover. This cancer-type specificity is consistent with TCGA pan-cancer analyses demonstrating that cell-of-origin patterns dominate molecular classification [19], with metabolically active organs such as kidney [21] and lung [22] exhibiting particularly pronounced epigenetic sensitivity to environmental exposures.

### 4.5. Biological Plausibility: The One-Carbon Metabolism Connection

The mechanistic link between OBS and DNA methylation is likely mediated through one-carbon metabolism, a critical metabolic pathway that provides methyl groups for DNA methylation. Our OBS included key micronutrients such as folate and vitamin B6, which are essential cofactors for the synthesis of S-adenosylmethionine (SAM), the universal methyl donor required for DNA methylation reactions [32,33]. Folate deficiency has been specifically linked to altered DNA methylation patterns and increased risk of colorectal carcinogenesis [32], while methyl donor micronutrients collectively modify DNA methylation and influence cancer outcomes [33].

Antioxidants and TET Enzymes: Furthermore, vitamin C, a major component of our antioxidant score, serves as an essential cofactor for Ten-eleven translocation (TET) enzymes, which catalyze the active demethylation of DNA through conversion of 5-methylcytosine (5-mC) to 5-hydroxymethylcytosine (5-hmC) [34]. This vitamin C-dependent regulation of the epigenome [34] provides a direct mechanistic explanation for the specific hypomethylation patterns we observed in individuals with high OBS scores.

The bidirectional nature of this relationship is further supported by evidence that oxidative stress itself can alter DNA methylation patterns. Environmental exposures inducing oxidative stress, such as metal-rich nanoparticles, have been shown to cause concurrent changes in oxidative markers and DNA methylation [35], demonstrating that the oxidative balance-methylation axis operates in both directions.

The distinct CpG signatures identified in our study likely reside in gene promoters or enhancers regulated by these nutrient-sensitive enzymatic pathways, representing oxidative stress-responsive epigenetic “hotspots” that integrate dietary and lifestyle exposures into stable molecular marks [32,33,34].

### 4.6. Strengths and Limitations

This study has several key strengths. First, the use of a multi-cohort design integrating population-based (KoGES) [18] and cancer genomic (TCGA) [19,20] data allows for the validation of population-level findings against pathological outcomes. Second, the development of the Hybrid Pi-score (HyPi) provides a novel, quantifiable metric to bridge the gap between epidemiological association studies and clinical genomics, building on established principles of significance scoring in genomic research [23,24]. Third, the comprehensive OBS calculation, incorporating 16 factors, offers a more holistic view of oxidative status than single-nutrient studies [5].

However, limitations exist. The KoGES methylation data is derived from blood leukocytes, while TCGA data comes from tissue samples. Although blood-derived DNA methylation can serve as a surrogate marker for systemic exposures, tissue-specific epigenetic effects may be masked or differ in magnitude [16,19]. The tissue-specific nature of DNA methylation [16] means that our blood-based signatures may underestimate organ-specific epigenetic changes. Additionally, the cross-sectional nature of the baseline analysis limits causal inference.

Future longitudinal studies should track methylation changes over time as individuals develop cancer. Furthermore, while we identified robust associations between OBS and DNA methylation patterns, future studies should integrate gene expression (mRNA) data to confirm whether these methylation changes result in functional transcriptional alterations and phenotypic consequences [16]. Multi-omic integration would provide a more complete picture of how oxidative balance influences cancer risk through epigenetic mechanisms.

## 5. Conclusions

In conclusion, our study provides the first comprehensive evidence of a robust, sex-specific epigenetic bridge linking the lifestyle-based Oxidative Balance Score to pan-cancer risk. By integrating large-scale population data (KoGES) [18] with cancer genomic profiles (TCGA) [19,20], we demonstrate that favorable oxidative balance is associated with specific DNA methylation signatures that exhibit strong directional consistency with cancer-associated epigenetic patterns, particularly in metabolically active organs such as kidney [21] and lung [22]. Our novel Hybrid Pi-score (HyPi) successfully quantifies the epigenetic impact of oxidative balance, incorporating principles of differential variability analysis [23,24] to bridge epidemiological associations with clinical cancer genomics. The complete absence of overlap in top OBS-associated CpG sites between males and females underscores the critical importance of sex-specific approaches in cancer prevention research [28,29], reflecting fundamental differences in how hormonal and oxidative stress pathways influence the epigenome [30,31].

Mechanistically, our findings support the role of one-carbon metabolism and vitamin C-dependent TET enzyme activity [32,33,34] in mediating the relationship between dietary antioxidants and DNA methylation patterns. The directional consistency observed in our Quad-plot analyses suggests that optimizing oxidative balance through diet and lifestyle modifications [5,6] may epigenetically modulate regulatory regions linked to oncogenic pathways or tumor suppressor genes.

The HyPi score emerges as a promising tool for identifying individuals at high epigenetic risk of cancer based on their oxidative balance profile, paving the way for precision nutrition and personalized prevention strategies. Future longitudinal studies should validate these findings prospectively, integrate multi-omic data (transcriptomics, proteomics) to confirm functional consequences of these methylation changes, and develop clinical translation pathways for implementing OBS-based risk stratification in cancer-screening programs.

## Data Availability

The R source code used for the analysis will be made available upon contacting the corresponding author.

## Supplementary Materials

The following supporting information can be downloaded at: https://www.mdpi.com/article/10.3390/antiox15030386/s1. Figure S1. Venn diagrams of the top 200 CpG sites most strongly associated with OBS in the KoGES cohort (HyPi score) between two sexes; Figure S2. Sexual dimorphism in epigenetic mechanisms linking OBS to cancer. A scatter plot comparing the cumulative epigenetic impact (Total HyPi × Pi Score) between males (x-axis) and females (y-axis) across various TCGA cancer cohorts. Each colored point represents a specific cancer type. The dashed diagonal line (y = x) indicates a theoretical shared mechanism. Points deviating from this diagonal demonstrate sex-specific epigenetic regulation, where the impact of OBS on cancer risk differs significantly between men and women; Figure S3. Heatmap of mean HyPi scores for Top 20 CpGs. A summary heatmap visualizing the mean Hybrid Pi-scores of the top 20 OBS-associated CpGs across different cancer types (x-axis) and comparison groups (NT, T1, T2 on y-axis) for males (left) and females (right). The color intensity reflects the average strength of the association, providing a high-level view of which clinical comparisons yield the strongest epigenetic signals; Figure S4. Variable contribution analysis of cancer types to OBS signals. Stacked bar charts quantifying the percentage contribution of individual cancer types to the total OBS-derived epigenetic signal in males (left) and females (right). The x-axis categorizes the data by clinical comparison type (NT, T1, T2). The colored segments within each bar represent different TCGA cancer types, as detailed in the legend. This figure identifies which specific cancers are most strongly epigenetically linked to oxidative balance in each clinical context.

## Abbreviations

The following abbreviations are used in this manuscript:

HyPi	Hybrid Pi-score
KoGES	Korean Genome and Epidemiology Study
OBS	Oxidative Balance Score
TCGA	The Cancer Genome Atlas
