# Supplementary material for: Epigenetic Bridge Between Oxidative Balance of Koreans and TCGA Pan-Cancer Risk: Sex-Specific DNA Methylation Signatures
Source: Antioxidants (Basel). 2026 Mar 19;15(3):386. doi: 10.3390/antiox15030386 (PMC13023465; doi:10.3390/antiox15030386)
Supplement: Supplementary file 1 [file antioxidants-15-00386-s001.zip › antioxidants-4120292-supplementary.pdf]

antioxidants

MDPI

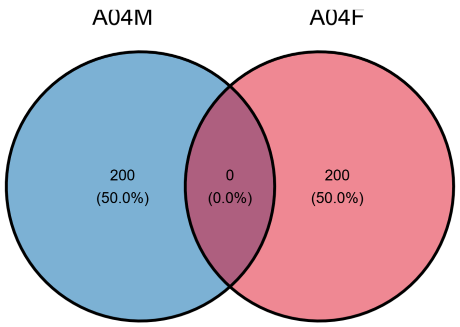

Supplementary Materials Figure S1. Venn diagrams of the top 200 CpG sites most strongly associated with OBS in the KoGES cohort (HyPi score) between two sexes.

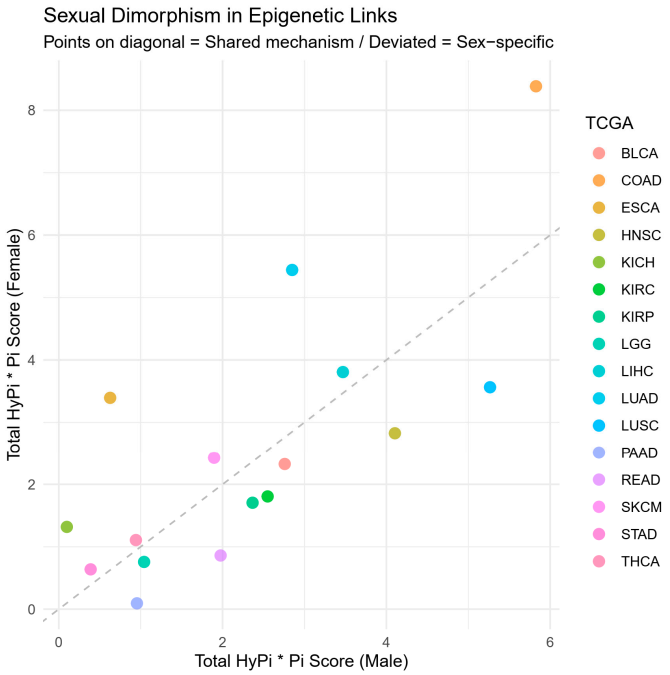

Supplementary Materials Figure S2. Sexual dimorphism in epigenetic mechanisms linking OBS to cancer. A scatter plot comparing the cumulative epigenetic impact (Total HyPi × Pi Score) between males (x-axis) and females (y-axis) across various TCGA cancer cohorts. Each colored point represents a specific cancer type. The dashed diagonal line (y=x) indicates a theoretical shared mechanism. Points deviating from this diagonal demonstrate sex-specific epigenetic regulation, where the impact of OBS on cancer risk differs significantly between men and women.

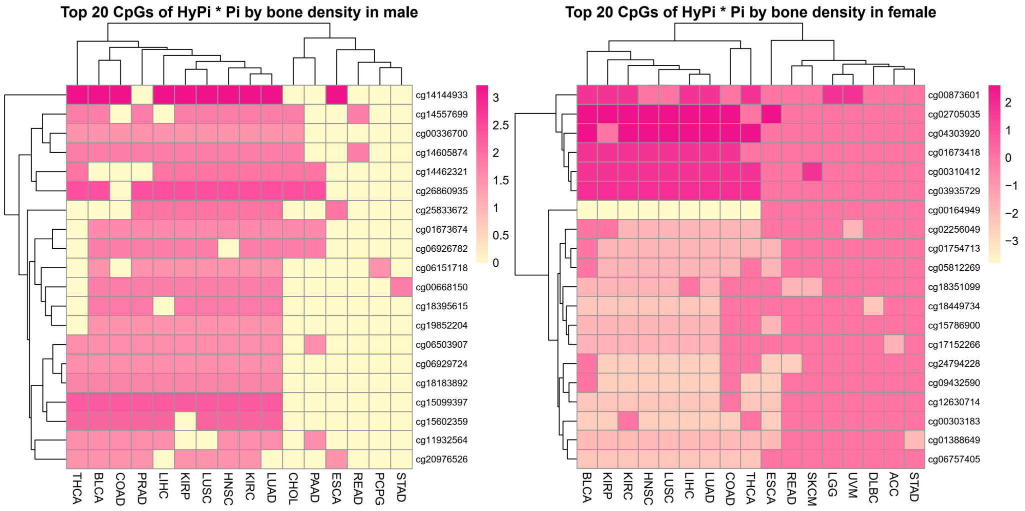

Supplementary Materials Figure S3. Heatmap of mean HyPi scores for Top 20 CpGs. A summary heatmap visualizing the mean Hybrid Pi-scores of the top 20 OBS-associated CpGs across different cancer types (x-axis) and comparison groups (NT, T1, T2 on y-axis) for males (left) and females (right). The color intensity reflects the average strength of the association, providing a high-level view of which clinical comparisons yield the strongest epigenetic signals.

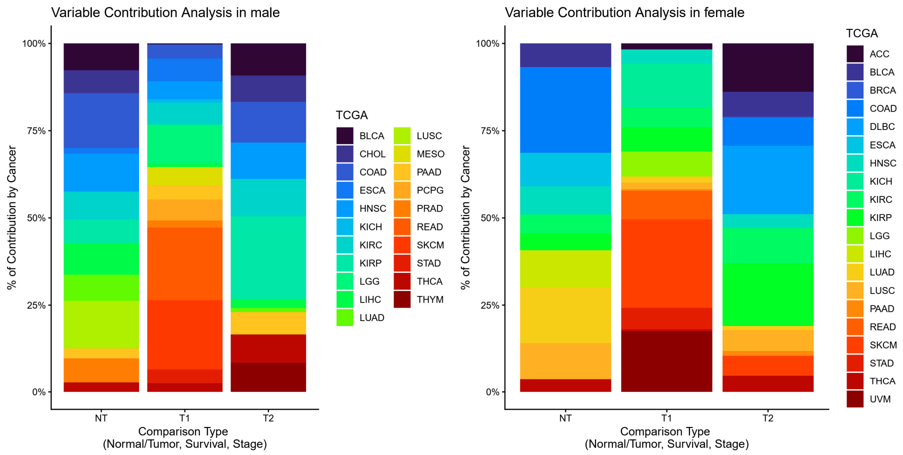

Supplementary Materials Figure S4. Variable contribution analysis of cancer types to OBS signals. Stacked bar charts quantifying the percentage contribution of individual cancer types to the total OBS-derived epigenetic signal in males (left) and females (right). The x-axis categorizes the data by clinical comparison type (NT, T1, T2). The colored segments within each bar represent different TCGA cancer types, as detailed in the legend. This figure identifies which specific cancers are most strongly epigenetically linked to oxidative balance in each clinical context.
